# Isoprene deters insect herbivory by priming plant hormone responses

**DOI:** 10.1126/sciadv.adu4637

**Published:** 2025-04-18

**Authors:** Abira Sahu, Mohammad Golam Mostofa, Yuan Xu, Bianca M. Serda, James O’Keefe, Thomas D. Sharkey

**Affiliations:** ^1^ Department of Energy Plant Research Laboratory, Michigan State University, East Lansing, MI, USA.; ^2^ Plant Resilience Institute, Michigan State University, East Lansing, MI, USA.; ^3^ Department of Environmental Health Sciences, University of Alabama, Birmingham, AL, USA.; ^4^ Department of Biochemistry and Molecular Biology, Michigan State University, East Lansing, MI, USA.; ^5^ Department of Chemistry, State University of New York College of Environmental Science and Forestry, Syracuse, NY, USA.; ^6^ Mass Spectrometry and Metabolomics Core, Michigan State University, East Lansing, MI, USA.

## Abstract

Isoprene, emitted by some plants, deters insect herbivory. However, the associated biochemical and physiological responses that confer herbivory resistance remain unknown. We used engineered isoprene-emitting (IE) and non-emitting (NE) control tobacco plants to interpret isoprene-mediated defense against herbivory in plants. Hornworm larvae raised on IE plants exhibited stunted growth compared to those raised on NE plants. Worms preferred to feed on NE rather than IE leaves, indicating deterrent effects of isoprene on insect feeding. Worm feeding induced a greater increase in jasmonic acid (JA), a crucial hormone for insect resistance, in IE leaves compared to that in NE leaves. Assimilation rates were stably maintained in IE plants, suggesting a protective role of isoprene in preserving photosynthetic efficiency during insect herbivory. Wound-induced increase in isoprene emission correlated with the elevation of key metabolites of the isoprene biosynthesis pathway. Our results highlight JA-priming functions of isoprene and provide insights into isoprene-mediated defense against insect herbivory.

## Teaser

Isoprene, emitted from plants to the atmosphere in large amounts, repels insect herbivory by increasing jasmonic acid in plants.

## INTRODUCTION

Plants encounter a variety of environmental stresses throughout their life cycle, with pest infestations being one of the common challenges. Annual crop loss to pest attacks ranges between 20 and 40% (*1*), resulting in a global economic loss of $70 billion. The yield reduction is further exacerbated by global warming because elevated temperatures accelerate insect metabolism, leading to increased consumption of plant tissues (*2*, *3*). Global yield loss to insects is anticipated to increase by 10 to 25% for each degree temperature rise (*4*). Pest attacks have a negative impact on several aspects of food security and economic viability including production costs and stability, distribution efficiency, crop nutritional value, and biomass quality (*5*). Hence, increased pest infestation necessitates greater pesticide application to mitigate yield loss, further deteriorating plant health and the environment. Therefore, it is crucial to find or develop plants with enhanced pest resistance for ensuring global food security and economic viability.

Synthesis and emission of volatile organic compounds (VOCs), particularly terpenes such as monoterpenes and sesquiterpenes, are a key defense strategy used by plants to counteract insect herbivory (*6*–*9*). In addition to these, many plants emit a hemiterpene, isoprene, which accounts for 50 to 60% of the total global nonmethane hydrocarbon emission from the biosphere (*10*). Once in the atmosphere, isoprene interacts with hydroxyl radicals to produce free radicals, which contribute to ozone and aerosol formation in the presence of nitrogen oxides (NO*_x_*) (*11*, *12*). On the other hand, it plays a crucial role in protecting plants from abiotic stresses including high temperature, drought, and ozone stress (*13*–*20*).

Isoprene is synthesized in leaf chloroplasts through the methyl-d-erythritol-4-phosphate (MEP) pathway (*21*). Glyceraldehyde 3-phosphate and pyruvate of the primary CO_2_-fixing reactions of photosynthesis, the Calvin-Benson cycle (CBC), serve as precursors for the synthesis of 1-deoxy-d-xylulose-5-phosphate (DXP), the first MEP metabolite (*21*). MEP undergoes a series of enzymatic reactions and ultimately produces isoprene from dimethylallyl diphosphate (DMADP) catalyzed by isoprene synthase (ISPS) (*21*). The biosynthesis of each molecule of isoprene is highly energy intensive, requiring 14 NADPH (reduced form of nicotinamide adenine dinucleotide phosphate) and 20 ATP (adenosine triphosphate) molecules (*21*). Presumably, this metabolic cost is offset by the benefits associated with isoprene in enhancing plant defense responses. Isoprene acts as a signaling molecule, modulating the plant transcriptome, proteome, and metabolome enhancing resilience against various environmental stresses (*22*–*24*). However, the role of isoprene in biotic stress tolerance and the associated underlying mechanistic pathways is largely unexplored.

Plants have evolved complex signaling mechanisms involving phytohormones, particularly jasmonic acid (JA), to defend against insect herbivory (*25*). Within 2 hours of pest infestation, there is a rapid increase in endogenous JA and its bioactive derivative, jasmonoyl isoleucine (JA-Ile) (*26*). Jasmonate-deficient mutants are more susceptible to insect feeding than wild-type plants, highlighting the crucial role of JA in insect resistance (*27*, *28*). Isoprene modulates the expression of several JA-biosynthetic genes in unstressed plants (*22*). Most isoprene-responsive genes contain several cis-regulatory elements that interact with transcription factors involved in JA signaling (*29*). Furthermore, genes involved in the biosynthesis of stress-related metabolites including glucosinolates, polyamines, oxylipins, apiose, and phenylpropanoids are up-regulated in isoprene-emitting (IE) plants (*22*). Isoprene also promotes phosphorylation of nitrate-induced (NOI) peptides (*23*). Genes of the NOI family are up-regulated in *Arabidopsis* plants after spider mite feeding (*30*). These results indicate the potential functions of isoprene in eliciting plant defense responses against insect herbivory. Tobacco hornworms (*Manduca sexta*) preferred feeding on non-emitting (NE) plants over IE plants (*31*), but there is a gap in our understanding of the mechanism(s) underlying isoprene-mediated plant defense responses to insect herbivory.

In this study, we investigated the underlying physiological and biochemical changes in IE plants during insect herbivory to understand why IE plants are more pest resistant. We used homozygous IE tobacco plants transformed with an *isoprene synthase* (*ISPS*) gene from *Populus alba* and the corresponding azygous (NE) control in this study. We identified the deterrent effect of isoprene on whitefly infestation and hornworm development in IE tobacco plants. We demonstrated that assimilation rates were not significantly affected in IE plants during worm feeding. We assessed changes in the hormone profile in IE leaves following insect feeding and wounding. We also evaluated wound-induced isoprene emission and its connection with MEP pathway and CBC metabolites in IE plants. Targeted metabolomics revealed that wounding induced an increase in pyruvate levels, a key substrate of the MEP pathway. This, in turn, led to elevated levels of several MEP metabolites including DXP, 2-*C*-methyl-d-erythritol-2,4-cyclodiphosphate (MEcDP), and 4-hydroxy-3-methylbut-2-enyl-diphosphate (HMBDP), ultimately resulting in enhanced isoprene emission from wounded leaves.

## RESULTS

### Isoprene deters whitefly infestation and hornworm herbivory

**Fig. 1.**
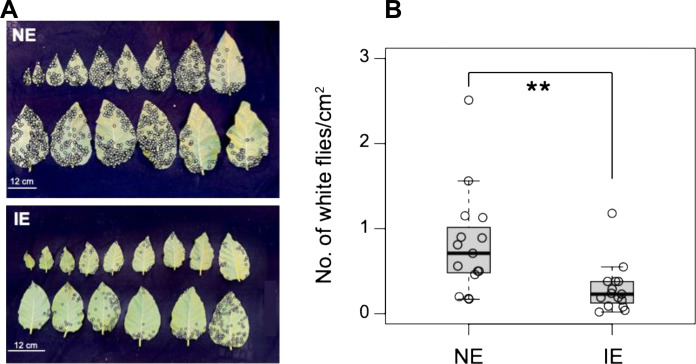
Effect of isoprene emission on white fly infestation. (**A**) White flies (shown by black circles) in IE and NE tobacco plants grown together in the same pot (*n* = 12 to 15 leaves). (**B**) Quantification of white fly infestation per square centimeter leaf area in NE and IE plants. Asterisks indicate a significant decline in whitefly infestation in IE leaves compared to that in NE leaves (*P* < 0.01; Student’s *t* test). Whiskers of the box plots represent 95% confidence interval.

**Fig. 2.**
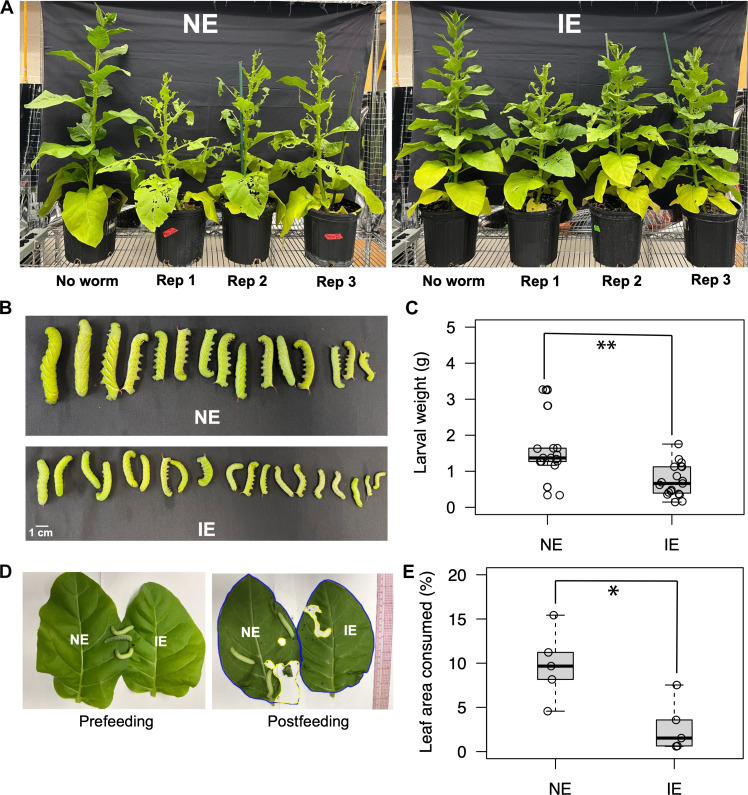
Effect of isoprene emission on insect herbivory and hornworm feeding preference study. (**A**) Comparison of leaf consumption by tobacco hornworms in NE and IE plants (*n* = 3). (**B** and **C**) Comparison of hornworm larval weight reared on IE and NE plants for 10 days (*n* = 15 to 18). Asterisks indicate significantly lower leaf consumption in IE leaves compared to that in NE leaves (*P* < 0.01; Student’s *t* test). (**D**) Comparison of leaf consumption by 10-day-old worms when given the choice between NE and IE leaves. (**E**) Quantification of leaf consumption 90 min after placing worms on a pair of IE and NE leaves (*n* = 4). The asterisk indicates significantly lower weight of worms feeding on IE leaves compared to those feeding on NE leaves (*P* < 0.05; Student’s *t* test). Whiskers of the box plots represent 95% confidence interval.

To assess the impact of isoprene emission on plant resistance to insect attack, transgenic IE tobacco plants (hereafter referred as IE plants) were grown alongside NE plants in the same pot. Insect infestation was quantified by counting the number of whiteflies per square centimeter leaf area of 8-week-old IE and NE plants (Fig. 1A). The number of whiteflies was significantly lower in IE plants than NE plants (Fig. 1B). To further evaluate the impact of isoprene emission on insect herbivory, an equal number of tobacco hornworm first instar larvae were placed on IE and NE plants and leaf area consumption was quantified after 10 days of worm feeding. The hornworms ate less of the leaves from the IE plants compared to those from the NE plants (Fig. 2A). The hornworms that fed on IE leaves exhibited growth reduction, leading to significantly lower larval weight compared to those feeding on NE leaves (Fig. 2, B and C).

### Isoprene influences hornworm feeding preference

An in vivo feeding preference study was conducted by placing third instar hornworm larvae on a pair of IE and NE leaves (Fig. 2D). After 90 min of feeding, the hornworms consumed significantly less of the IE leaves compared to the NE leaves (Fig. 2E). Real-time video monitoring revealed that, although the worms crawled over the IE leaf, they refrained from feeding on it and preferred to feed on the NE leaf instead (movie S1). Upon closely reviewing the video, we noticed that one worm spent 26% of the recorded time on the IE leaf; however, it did not consume any part of the leaf. After staying on the IE leaf for 7 to 8 min, the hornworm eventually moved toward the NE leaf and began to feed on it. To further evaluate their feeding preference, first instar hornworm larvae were placed in a box containing a pair of IE and NE leaves (fig. S1A). After 7 days of feeding, consumption of the IE leaf was significantly lower compared to that of the NE leaf (fig. S1B). In addition, worms recovered from the IE leaf after 7 days exhibited significantly lower larval weight compared to those on the NE leaf (fig. S1C).

### Worm feeding effects on photosynthetic biochemistry

**Fig. 3.**
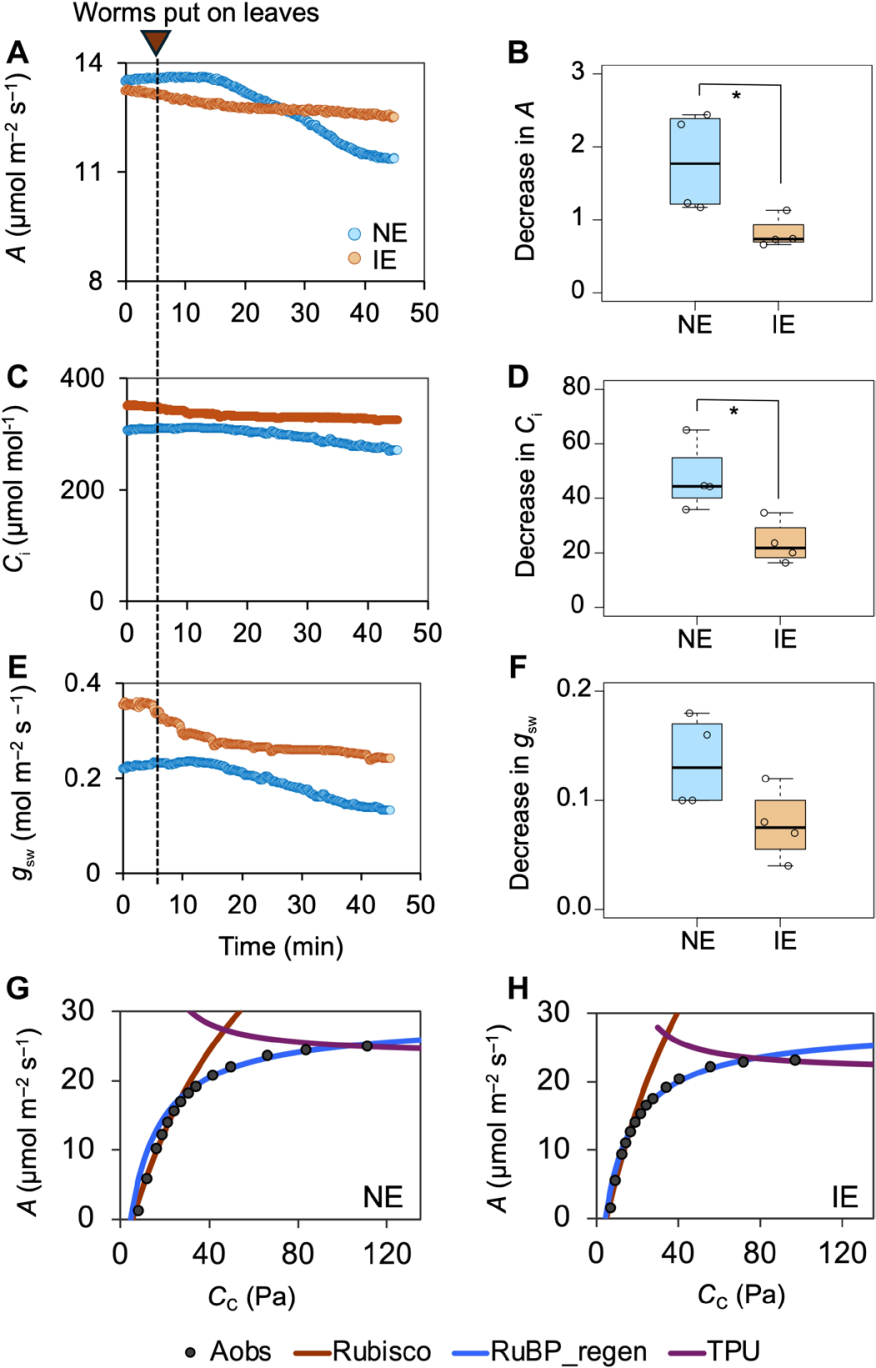
Effect of worm feeding on photosynthesis (*A*), intercellular CO_2_ concentration (*C*_i_), and stomatal conductance (*g*_sw_) in NE and IE leaves. (**A** to **C**) Changes in *A*, *C*_i_, and *g*_sw_ recorded during worm feeding in NE and IE leaves. (**D** to **F**) Comparison of absolute changes in *A*, *C*_i_, and *g*_sw_ pre- and postfeeding in NE and IE leaves. The decline in *A* and *C*_i_ is significantly lower in IE leaves compared to NE leaves (*P* < 0.05; Student’s *t* test). Whiskers of the box plots represent 95% confidence interval. (**G** and **H**) Representative *A*/*C*_i_ curves made using the dynamic assimilation technique in NE and IE leaves during worm feeding. Black filled circles represent the measured CO_2_ assimilation rates (Aobs), the red line represents the fitted rubisco activity, the blue line represents RuBP regeneration, and the purple line shows the TPU limitation. No TPU limitation was observed during worm feeding.

To investigate the effect of insect herbivory on photosynthesis, gas exchange features including assimilation rate (*A*), intercellular CO_2_ concentration (*C*_i_), and stomatal conductance (*g*_sw_) were recorded in NE and IE leaves during worm feeding. Insect feeding induced stomatal closure (lower *g*_sw_), resulting in decreased *C*_i_ and *A* in the undamaged areas of both IE and NE leaves (Fig. 3, A to C, and fig. S2, A to C). However, IE leaves showed significantly lower reduction in *A* than NE leaves (Fig. 3D). The milder effect of herbivory on *A* was consistent with a smaller decrease in *C*_i_ in worm-fed IE leaves compared to that in NE leaves (Fig. 3, E and F). *A*/*C*_i_ response curves revealed that this distinct photosynthetic response in IE leaves was unrelated to differences in rubisco activity, electron transport, and triosephosphate use limitation because these parameters were similar between NE and IE leaves (Fig. 3G and table S1), further supporting stomatal-mediated photosynthesis decline.

### Hornworm feeding effects on phytohormones

**Fig. 4.**
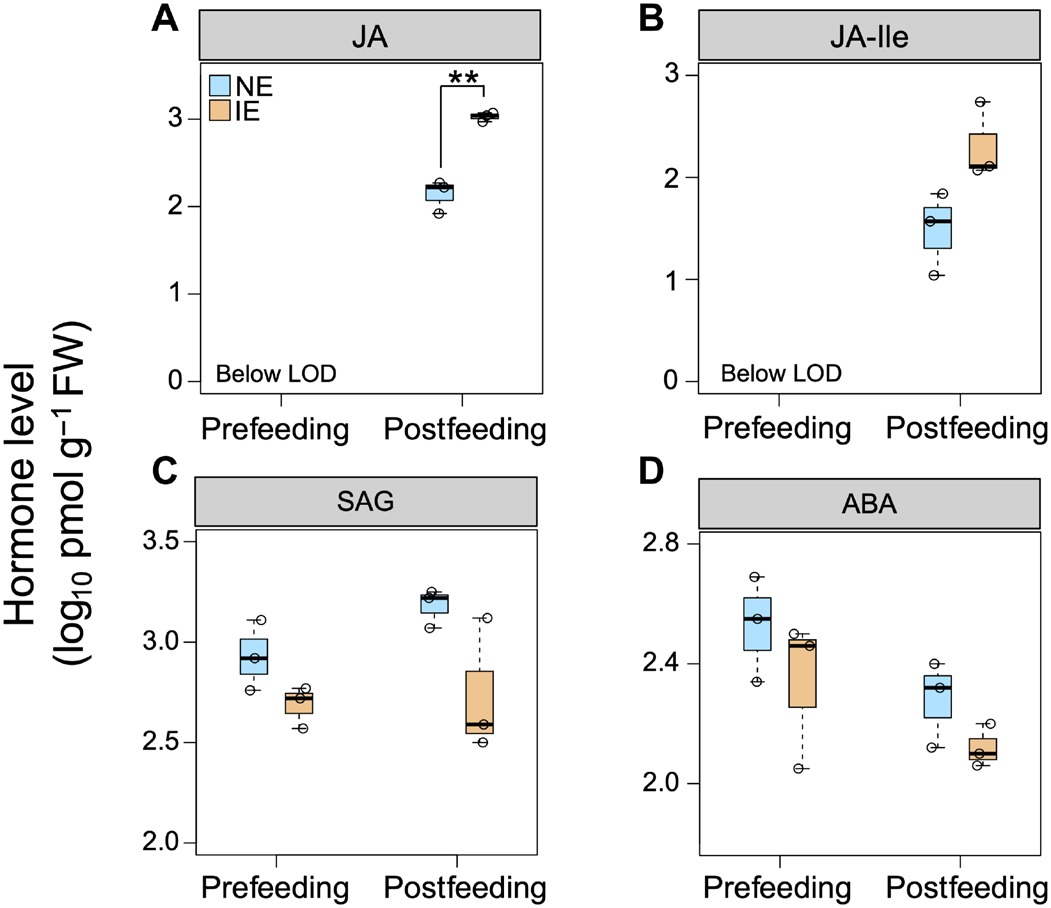
Change in hormone levels in NE and IE leaves after worm feeding. Hormones were quantified in leaves 1 hour postfeeding (*n* = 3). (**A**) The postfeeding JA level was significantly higher in IE leaves compared to NE leaves (*P* < 0.01; Student’s *t* test). Changes in (**B**) JA-Ile, (**C**) SAG, and (**D**) ABA levels between NE and IE lines were not statistically significant. Whiskers of the box plots represent 95% confidence interval. Abbreviations: LOD, limit of detection; FW, fresh weight.

Levels of JA, JA-Ile, salicylic acid (SA), salicylic acid glucoside (SAG), and abscisic acid (ABA) were quantified in IE and NE leaf samples collected after 1 hour of worm feeding. While undetectable in prefeeding leaves, the JA level was significantly elevated in the leaves of both IE and NE plants postfeeding. Notably, the increase in JA in IE leaves was significantly higher than that in NE leaves (Fig. 4A). JA-Ile showed a similar trend, although the increase was not statistically significant (*P* = 0.06; Fig. 4B). SA was not detected in any sample, both pre- and postfeeding. Moreover, there were no significant differences in SAG and ABA levels between NE and IE leaves before and after hornworm feeding (Fig. 4, C and D). To determine whether this hormone response persisted over the long term, leaf samples were collected from NE and IE plants 10 days after insect feeding. For control, leaf samples were collected from IE and NE plants of the same age that were never exposed to hornworms. Notably, all the studied hormones were similar after long-term herbivory, except for ABA. The ABA level was significantly higher in hornworm-fed IE leaves (fig. S3).

### Change in isoprene emission, metabolic, and phytohormone levels in mechanically wounded leaves

**Fig. 5.**
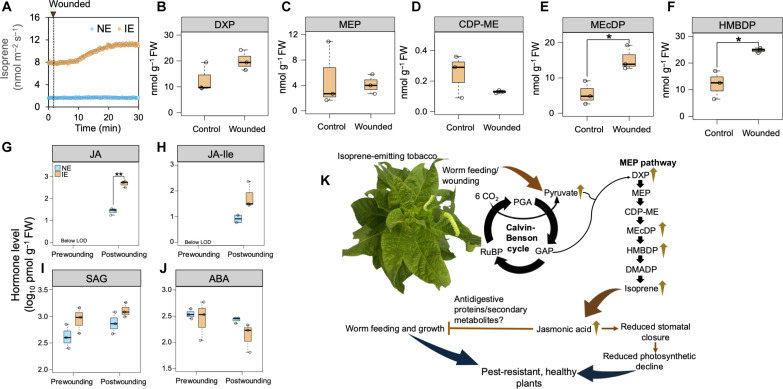
Effect of mechanical wounding on isoprene emission, MEP pathway metabolites, and hormones. (**A**) Change in isoprene emission in NE and IE leaves after wounding. (**B** to **F**) Change in MEP pathway metabolites in wounded IE leaves compared to that in unwounded (control) leaves. The increase in HMBDP and MEcDP is significantly higher after wounding (*P* < 0.05; Student’s *t* test). (**G** to **J**) Change in hormone levels in NE and IE leaves after mechanical wounding. Hormones were quantified in leaves 1 hour postwounding (*n* = 3). Asterisks indicate significantly higher JA levels in IE leaves postwounding compared to those in NE leaves (*P* < 0.01; Student’s *t* test). Whiskers of the box plots represent 95% confidence interval. (**K**) Mechanism of isoprene-mediated pest resistance. Increased JA levels in IE plants may lead to elevated levels of defense compounds, making IE plants more resistant to insect herbivory. Abbreviations: PGA, 3-phosphoglycerate; GAP, glyceraldehyde 3-phosphate.

Part of the damage done to leaves by insect chewing is mechanical damage to leaf tissue. To evaluate the impact of mechanical wounding on isoprene emission, IE leaves were wounded with forceps once photosynthesis and isoprene emission from the leaves were stable. Isoprene emission started rising within 1 min of wounding and continued to increase steadily until it became stable after 30 min (Fig. 5A).

To explore the underlying metabolic changes responsible for the wound-induced increase in isoprene emission, levels of MEP pathway metabolites were quantified in both wounded and unwounded (control) IE leaves (Fig. 5, B to F). Wounded leaves showed a significant increase in MEcDP and HMBDP levels compared to the control (Fig. 5, E and F). DXP displayed a similar trend, although the increase was not statistically significant (*P* = 0.15; Fig. 5B). No significant changes were observed in MEP and 4-cytidine-5′-diphospho-2-*C*-methyl-d-erythritol (CDP-ME) levels after wounding (Fig. 5, D and E). While DMADP peaks were not detectable by liquid chromatography–tandem mass spectrometry (LC-MS/MS) in this experiment, significant increases in the downstream metabolites like HMBDP and MEcDP presumably contributed to the elevated isoprene emission in response to wounding.

Given the observed trend of increased DXP in the wounded samples, we further analyzed postwounding changes in CBC metabolite levels to assess whether there was an enhanced substrate supply from the CBC to MEP pathway. The level of pyruvate, a key substrate for the MEP pathway, was significantly elevated in wounded IE leaves compared to the control (fig. S4). This change was not observed in the NE leaves. Other CBC metabolites that showed increases specifically in IE leaves after wounding included glucose 6-phosphate/fructose 6-phosphate, ribose 5-phosphate/ribulose 5-phosphate/xylulose 5-phosphate, and 6-phosphogluconate (fig. S4).

To assess whether the hormone response was similar between leaves damaged by insect feeding and mechanical wounding, the levels of JA, JA-Ile, SA, SAG, and ABA were quantified in leaf samples collected 1 hour after wounding. The hormone profiles in the wounded samples resembled the pattern of insect-fed leaves, with JA showing a significant increase in IE leaves compared to that in NE leaves (Fig. 5G). JA-Ile exhibited a similar trend, although the increase in IE leaves was not statistically significant at the 0.05 threshold (Fig. 5H). SA was undetectable in all samples, while SAG and ABA levels did not differ significantly between NE and IE leaves before or after wounding (Fig. 5, I and J).

## DISCUSSION

Plant-derived VOCs can help mitigate pest damage either directly by producing toxic, antinutritional, and repellent compounds or indirectly by attracting natural predators of the herbivore (*32*). The interaction between plants and insects involves complex signaling mechanisms mediated by various signaling molecules including phytohormones. JA is a primary defense-related phytohormone that triggers plant immunity against herbivory via a multistep signaling cascade (*25*). Insect herbivory induced a stronger increase in JA levels in IE plants than in NE plants (Fig. 4A). Therefore, isoprene possibly activates downstream defense signaling pathways by priming the JA response during pest infestations in IE plants.

Plants release up to 2% of the assimilated carbon as isoprene, and under extreme environmental conditions like heat stress (e.g., 42°C), the carbon used for isoprene synthesis can surpass 25% (*21*, *33*) of recently fixed carbon. This substantial allocation of resources toward isoprene biosynthesis suggests that isoprene offers important benefits. This study demonstrates that isoprene plays an important role in eliciting plant defense responses against pest infestations. Isoprene effectively deterred hornworm feeding (Fig. 2, A to C). Isoprene has also been shown to deter parasitic wasps in a tritrophic response (*34*). However, in that case, isoprene interfered with parasitic wasps that would protect the plant, which could result in more damage of IE plants rather than less damage.

The deterrent effect of isoprene toward worm feeding was further supported by an in vivo feeding preference study that demonstrated that hornworms avoided feeding on IE leaves (Fig. 2, D and E, and movie S1), consistent with the findings of Laothawornkitkul *et al.* (*31*). We did not observe any influence of IE plants on NE plants growing in the same pot in resisting whitefly infestation (Fig. 1) or when IE and NE leaves were in direct contact during hornworm growth (fig. S1). This could be due to the diluted level of isoprene in the air being insufficient to activate defense responses in neighboring NE plants. This contrasts with the function of other VOC signals such as (*E*)-4,8-dimethyl-1,3,7-nonatriene, a homoterpene, which can induce a defense response in neighboring, untreated plants independently of JA accumulation (*35*).

Physiological responses of plants to insect herbivory result in a more substantial reduction of photosynthetic capacity in undamaged leaf tissue than the direct loss of photosynthetic tissue to chewing damage (*36*). The decline in CO_2_ assimilation rates in the unwounded leaf area was less pronounced in the IE tobacco plants after worm feeding (Fig. 3, A and D), similar to the response of aphid-resistant barley and wheat cultivars (*37*, *38*). We further investigated the underlying mechanism of this differential photosynthetic response. The decrease in rubisco activation or ribulose 1,5-bisphosphate (RuBP) regeneration sometimes leads to reduced photosynthesis following pest attack (*38*). However, changes in these parameters between IE and NE tobacco plants remained indistinguishable during worm feeding (Fig. 3G and table S1). Rather, reduced stomatal closure in response to insect herbivory contributed to a smaller decline in photosynthesis in the IE plants.

A strong correlation between reduced stomatal conductance and decline in photosynthesis was observed in tobacco leaves exposed to hornworm oral secretions (*39*). The reduced stomatal closure may be associated with increased JA levels in IE plants, because application of coronatine (a JA-Ile analog) has been shown to delay stomatal closure (*40*). In addition, the complete loss of photosynthetic responses in *aoc* (*allene oxide cyclase*) mutants to insect oral secretions highlights the role of JAs in regulating stomatal conductance–mediated carbon assimilation changes (*39*).

Transcriptome profiling revealed that isoprene up-regulates the expression of JA-biosynthetic genes, including *lipoxygenases* and *12-oxophytodienoate reductase 3* (*OPR3*) in unstressed IE transgenic *Arabidopsis* and tobacco plants (*22*). In addition, 80 to 100% of genes up-regulated by isoprene treatment contain cis-regulatory elements like MYC recognition site, E-box, W-box, and GATA box, which interact with various transcription factors involved in JA signaling (*29*). These gene expression changes, along with extensive phosphoproteomic changes, were induced by isoprene fumigation in unstressed plants (*22*, *23*, *41*). Moreover, there was substantial overlap in the differentially expressed genes between isoprene-fumigated plants and those engineered to emit isoprene (*22*). Therefore, we propose that isoprene itself, rather than some precursor metabolite of the MEP pathway, is the active factor that triggers JA-mediated responses in the transgenic IE lines. Furthermore, worm feeding in tobacco plants results in jasmonate-induced accumulation of secondary metabolites such as nicotine, caffeoylputrescine, and 17-hydroxygeranyllinalool diterpenoid glycosides and increased levels of antidigestive proteins like polyphenol oxidase, trypsin proteinase inhibitors, and threonine deaminase (*42*). These results, together with the current data, suggest that isoprene induces stronger JA responses by increased JA synthesis and/or signaling, possibly leading to the accumulation of insect-growth inhibitory metabolites that makes IE plants more resistant to insect herbivory.

SA is the other key phytohormone that regulates plant defense responses to insect herbivory (*43*). It engages in complex cross-talk with JA to modulate these defense mechanisms (*44*). MEcDP, an intermediate metabolite of the MEP pathway, functions as a positive regulator of the SA signaling pathway (*45*–*47*). However, MEcDP-induced increases in SA levels do not interfere with the gene expression associated with JA signaling upon insect herbivory (*48*). In the current study, although MEcDP levels increased upon wounding, SA levels remained undetectable in wounded or insect-fed leaves, while JA levels increased upon wounding and insect herbivory. Therefore, we propose that the increase in JA levels upon worm feeding in IE plants is driven by isoprene rather than MEcDP.

Transgenic IE plants showed widespread changes in differential gene expression involved in various stress resilience pathways including increased resistance to herbivory and wounding (*22*). Other than up-regulation of JA biosynthesis and signaling, isoprene altered the expression of genes involved in phenylpropanoid, apiose, callose, and cellulose synthesis (*22*). Certain phenylpropanoids, such as tannins and flavonoids, disrupt the growth of herbivores, while lignin increases tissue toughness to deter herbivore feeding (*49*). The incorporation of apiose into cell wall polysaccharides can enhance the structural complexity and rigidity of the cell wall, making it more difficult for herbivorous insects to penetrate and digest plant tissues (*50*). Similarly, callose deposition serves as a physical barrier, preventing phloem ingestion by insects (*51*). Notably, these gene expression changes were observed in unstressed plants. However, whether the levels of these secondary metabolites are altered in response to insect herbivory in IE plants remains to be determined.

On the other hand, exogenous JA affects isoprene emission, although the response varies between species. JA treatment was found to increase both local and systemic isoprene emissions from *Populus tremuloides* because of an increase in newly fixed carbon (*52*). However, isoprene emission declined in *Ficus septica* after JA spraying, which correlated with reduced *ISPS* gene expression and protein levels (*53*). JA also plays an important role in the growth-defense trade-off in plants. Degradation of jasmonate ZIM-domain (JAZ) proteins (*54*) in the presence of JA subsequently activates MYC transcription factors, which negatively regulate leaf growth (*55*, *56*). IE plants exhibit stunted growth relative to NE plants, suggesting a connection of isoprene in growth-defense trade-off. It is likely that isoprene-induced higher JA levels in IE plants might activate the JAZ-MYC regulatory network, leading to the repression of growth-related pathways (*22*). However, the exact regulatory point of JA and isoprene interaction in mediating growth-defense trade-off is still unclear, warranting further research at the genetic level.

The emission of various VOCs such as monoterpenes, sesquiterpenes, methyl salicylate, methyl jasmonate, and green leaf volatiles from plants typically increases in response to insect herbivory (*57*). However, isoprene emission from oak, a natural emitter, declined when the leaves experienced 40 to 50% chewing damage (*58*). Because the change in isoprene emission from IE tobacco plants upon short-term insect herbivory was unnoticeable, mechanical wounding was conducted to elicit a stronger response. Enhanced isoprene emission was detected from the undamaged part of the wounded IE leaves (Fig. 5A), suggesting its stress-responsive nature.

Unlike the transient isoprene burst detected in *Phragmites australis* after leaf cutting (*58*), IE tobacco leaves showed a sustained isoprene emission for at least 20 min postwounding (Fig. 5A). The mechanism of this persistent isoprene emission was revealed from the profiling of MEP pathway– and photosynthesis-related metabolites. The elevated levels of several MEP pathway metabolites in wounded IE leaves coincided with the enhanced isoprene emission upon wounding (Fig. 5, B, E, and F). We detected a significant increase in MEcDP pool upon wounding, consistent with an earlier study (*59*). Furthermore, an increase in pyruvate levels in the wounded leaves (fig. S4) indicates an enhanced substrate supply from the CBC to MEP pathway in response to wounding. This change, however, was not observed in NE plants, supporting the notion that the increased defense response of IE plants against herbivory resulted from a coordinated metabolic shift to produce more isoprene. Furthermore, postwounding increases in 6-phosphogluconate and pentoses (fig. S4) indicate an increased rate of the oxidative pentose phosphate pathway, which is crucial for supplying reducing power and intermediates important for mounting a defense response against stresses including insect herbivory (*60*).

In summary, wounding triggered a rapid and sustained increase in isoprene emission from damaged leaves driven by shifts in carbon flow from the CBC to MEP pathway, as indicated by metabolomic data. In addition, this study reveals the connection of isoprene with the JA response during insect herbivory, thus suggesting a potential mechanism of isoprene-mediated defense from pest infestations in plants (Fig. 5K). Elevated JA levels may result in increased accumulation of defense compounds such as secondary metabolites and/or antidigestive proteins that make IE plants more resistant to insect herbivory. Given its deterring effect, the isoprene emission trait might be genetically introduced into NE crop plants to combat insect herbivory. Enabling plants to defend against pests via isoprene production could reduce reliance on chemical pesticides, fostering sustainable agricultural practices. In addition, minimizing crop loss because of insect herbivory will ensure economic stability for the farmers. Thus, harnessing the protective roles of isoprene may offer a viable strategy for pest management in plants amid climate instability.

## MATERIALS AND METHODS

### Plant growth

The IE transgenic tobacco (*Nicotiana tabacum*, “Samsun”) line and the NE control were obtained from Claudia Vickers, University of Queensland (*17*). The NE and IE plants used in this study correspond to line 32, previously identified as the highest isoprene producer among the IE tobacco lines (*17*). The information about plasmid design, vector construction, transformation, and selection of the lines are described by Vickers *et al.* (*17*). The IE and NE lines were grown in separate greenhouses in the Michigan State University Plant Research Laboratory. Each greenhouse had these conditions: 16-hour photoperiod at a light intensity of 400 to 500 μmol m^−2^ s^−1^, day/night temperatures of 25° to 27°C/20° to 22°C, and 60 to 65% humidity. The plants were grown from seeds in Suremix growing medium (Michigan Grower Products, Galesburg, MI). Individual seeds were first sown onto separate trays. Fourteen days after germination, seedlings were transplanted into small 3.5-liter pots to ensure seedling survival (five seedlings per pot). Fourteen days after transplantation when the seedlings are stable, they were transferred to large 7-liter pots (one plant per pot). The plants were watered with deionized water for 2 days and one-half-strength Hoagland nutrient solution for 5 days (*61*). Plants were used for experiments when they were 6 to 8 weeks old before they developed flowers and seeds.

### Insect growth and feeding assays

Tobacco hornworm eggs (Carolina Biological Supply, Burlington, NC) were incubated for 2 days in a glass box with detached tobacco leaves at room temperature. After the eggs hatched, the first instar larvae were transferred to 6-week-old plants with a paint brush. The plants were then kept in a growth chamber (16-hour photoperiod, mean light intensity of 400 to 500 μmol m^−2^ s^−1^, and day/night temperatures of 25° to 27°C/20° to 22°C). Larval weights were measured using an analytical balance, and consumed leaf areas were analyzed using Fiji (*62*).

### Phytohormone extraction and measurement by LC-MS/MS

For the short-term feeding assay, third instar larvae were placed on NE and IE leaves of 6-week-old plants. After 1 hour of feeding, leaf discs were collected using a freeze clamp. For the long-term feeding assay, first instar larvae were placed on NE and IE leaves of 6-week-old plants and allowed to feed for 10 days. For mechanical wounding, a part of the leaf outside the gas exchange chamber was wounded with forceps and leaf discs within the gas exchange chamber were collected after 1 hour. Frozen leaf discs were ground into a fine powder using a tissue homogenizer (Mixer Mill MM301, Retsch, Newton, PA). Then, 800 μl of ice-cold extraction buffer [80:20 (v/v) methanol:water, 0.1% formic acid, and butylated hydroxytoluene (0.1 g/liter)] was added to the ground plant material. Labeled internal standards (100 nM) including SA-^13^C_6_ (Santa Cruz Biotechnology, sc-220088), ABA-d_6_ (Toronto Research Chemicals, A110002), and JA-d_5_ (Cayman Chemical, 29076) were added to the extraction buffer. The tubes were vortexed and placed on a rocking platform at 4°C for 24 hours. On the next day, the samples were vortexed to mix and then centrifuged at 12,000*g* for 10 min at 4°C. Then, 400 μl of supernatant was transferred to centrifugal filter units (PALL Nanosep with a 0.2-μm polytetrafluoroethylene membrane) and centrifuged at 5000*g* for 1 min at room temperature. The flow-through was collected and aliquoted into 2-ml glass vials with inserts for LC-MS/MS analysis. Samples were analyzed by high-performance liquid chromatography after extraction. The hormones were separated using the Acquity UPLC BEH C18 column (2.1 by 50 mm, 1.7 μm) fitted on a Xevo TQ-XS mass spectrometer. Chromatographic separation used a multistep gradient with mobile phase A (water + 0.1% formic acid) and mobile phase B (acetonitrile): 0 to 0.5 min, 95% A; 0.5 to 10 min, 95 to 70% A; 10 to 11 min, 70 to 5% A; 11 to 13 min, 5% A; 13 to 13.01 min, 5 to 95% A, 13.01 to 15 min, 95% A, at a flow rate of 0.5 ml min^−1^. The column temperature was maintained at 40°C. The source temperature was maintained at 150°C, and the desolvation temperature was set to 400°C.

### Gas exchange measurements

Measurements of photosynthesis (*A*), intercellular CO_2_ concentration (*C*_i_), and stomatal conductance (*g*_sw_) in NE and IE leaves were taken using a LI-COR 6800 portable photosynthesis system (LI-COR Biosciences, Lincoln, NE). A fully expanded mature leaf was clamped to a 6-cm^2^ chamber of the LI-COR 6800 and maintained under a CO_2_ concentration of 420 μmol mol^−1^, a light intensity of 1000 μmol m^−2^ s^−1^, a temperature of 28°C, and a water vapor pressure difference of 1.3 kPa. Photosynthesis was allowed to stabilize, which took between 30 min and 1 hour. Once photosynthesis was stable, three to five third instar hornworm larvae were placed on a part of the leaf outside the chamber and photosynthetic measurements were recorded every 5 s for 45 min during worm feeding.

### *A*/*C*_i_ curve measurements

Representative *A*/*C*_i_ curves were generated using the dynamic assimilation technique in both NE and IE leaves during worm feeding. These curves were obtained using a LI-COR 6800 portable photosynthesis system (LI-COR Biosciences, Lincoln, NE) under a light intensity of 1000 μmol photons m^−2^ s^−1^, a leaf temperature of 28°C, and a vapor pressure difference of 1.3 kPa. The sequence of reference CO_2_ concentrations used was 50, 100, 200, 300, 350, 400, 450, 500, 550, 600, 700, 800, 1000, 1200, and 1500 μmol mol^−1^, as outlined by Sharkey (*63*). *A*/*C*_i_ curves from four replicates were fitted using the routine described by Sharkey (*64*). Photosynthesis and *A*/*C*_i_ curve parameters including the rubisco carboxylation capacity (*V*_cmax_), the electron transport capacity (*J*), the triose phosphate use (TPU) capacity, respiration in the light (*R*_L_), and mesophyll conductance to CO_2_ transfer (*g*_m_) were estimated using the method described by Sharkey (*64*). In addition, the proportion of carbon exported from photorespiration as glycine (α_g_) or serine (α_s_) was estimated using equations described by Busch *et al.* (*65*).

### Isoprene measurement in wounded leaves

Isoprene emission measurements were conducted using a fast isoprene sensor (FIS; Hills Scientific, Boulder, CO). A fully expanded mature leaf of IE plant was clamped to a 6-cm^2^ chamber of the LI-COR 6800. The leaf was allowed to equilibrate under the following conditions: light intensity of 1000 μmol m^−2^ s^−1^ (50% blue light and 50% red light), temperature of 30°C, CO_2_ concentration of 420 μmol mol^−1^, and water vapor content of 22 to 26 mmol mol^−1^ depending on laboratory room temperature. Then, a part of the leaf outside the chamber was wounded with forceps. Exhaust air from the LI-COR 6800 was fed into the FIS for isoprene measurements. The flow rate in the LI-COR 6800 was set at 500 μmol s^−1^, and the FIS flow rate was set such that it drew sample air from the LI-COR 6800 at 420 μmol s^−1^. An isoprene standard (3.225 parts per million) was used for the FIS calibration. Isoprene emission measurements were logged every 5 s.

### MEP pathway metabolite extraction and measurement by LC-MS/MS

Leaf discs were collected using a freeze clamp 20 min after wounding with forceps. MEP pathway metabolites were extracted using the protocol described by Sahu *et al.* (*66*). Frozen leaf discs were ground into a fine powder using a tissue homogenizer (Mixer Mill MM301, Retsch, Newton, PA) and extracted for 15 min using 300 μl of ice-cold extraction buffer containing 3:1:1 acetonitrile:isopropanol:20 mM ammonium bicarbonate, adjusted to pH 10 with ammonium hydroxide. The samples were then centrifuged at 14,000*g* for 10 min. The supernatant was transferred to 2-ml glass vials with glass inserts and analyzed by LC-MS/MS immediately following the extraction. Standards of the MEP pathway metabolites including DXP, MEP, CDP-ME, MEcDP, and HMBDP (Echelon Biosciences, Logan, UT) were separated using InfinityLab Poroshell 120 HILIC-Z, P column (2.1 by 100 mm, 2.7 μm with column ID) fitted on a Xevo TQ-XS mass spectrometer. The column temperature was maintained at 25°C. The mobile phase consisted of 20 mM ammonium bicarbonate adjusted to pH 10.0 with ammonium hydroxide and acetonitrile. Negative mode electrospray ionization was used with the following settings: capillary voltage of 1.00 kV, source temperature of 150°C, and desolvation temperature of 400°C.

### CBC metabolite extraction and measurement by ion-pair chromatography–tandem mass spectrometry

For the analysis of phosphorylated intermediates in the CBC, metabolites were extracted from rapidly frozen tissues using the protocol previously described by Xu *et al.* (*67*). Mass spectrometry analyses were performed following the methods outlined in earlier studies (*68*, *69*). Ion-pair chromatography–tandem mass spectrometry was performed using an Acquity UPLC pump system (Waters, Milford, MA) coupled with a Waters Xevo TQ-S UPLC/MS/MS (Waters, Milford, MA). Metabolites were separated on an Acquity UPLC BEH C18 Column (2.1 by 50 mm; Waters, Milford, MA) at 40°C. Chromatographic separation used a multistep gradient with mobile phase A [10 mM tributylamine in 5% (v/v) methanol] and mobile phase B (methanol): 0 to 1 min, 95 to 85% A; 1 to 6 min, 65 to 40% A; 6 to 7 min, 40 to 0% A; 7 to 8 min, 0% A; 8 to 9 min, 100% A, at a flow rate of 0.3 ml min^−1^. The source temperature was maintained at 120°C, and the desolvation temperature was set to 350°C. Nitrogen served as the sheath and auxiliary gas, with collision gas (argon) set to 1.1 mtorr. Gas flow values for desolvation and cone were adjusted to 800 and 50 liters/hour, respectively. The scan time was 0.1 ms.
