# Supplementary material for: Isoprene deters insect herbivory by priming plant hormone responses
Source: Sci Adv. 2025 Apr 18;11(16):eadu4637. doi: 10.1126/sciadv.adu4637 (PMC12007590; doi:10.1126/sciadv.adu4637)
Supplement: Supplementary file 1 — Figs. S1 to S4 Table S1 Legend for movie S1 [file sciadv.adu4637_sm.pdf]

Science Advances
AAAS

Supplementary Materials for

Isoprene deters insect herbivory by priming plant hormone responses

Abira Sahu *et al.*

Corresponding author: Mohammad Golam Mostofa, mmostofa@esf.edu; Thomas D. Sharkey, tsharkey@msu.edu

Sci. Adv. **11**, eadu4637 (2025)
DOI: 10.1126/sciadv.adu4637

The PDF file includes:

Figs. S1 to S4
Table S1
Legend for movie S1

Other Supplementary Material for this manuscript includes the following:

Movie S1

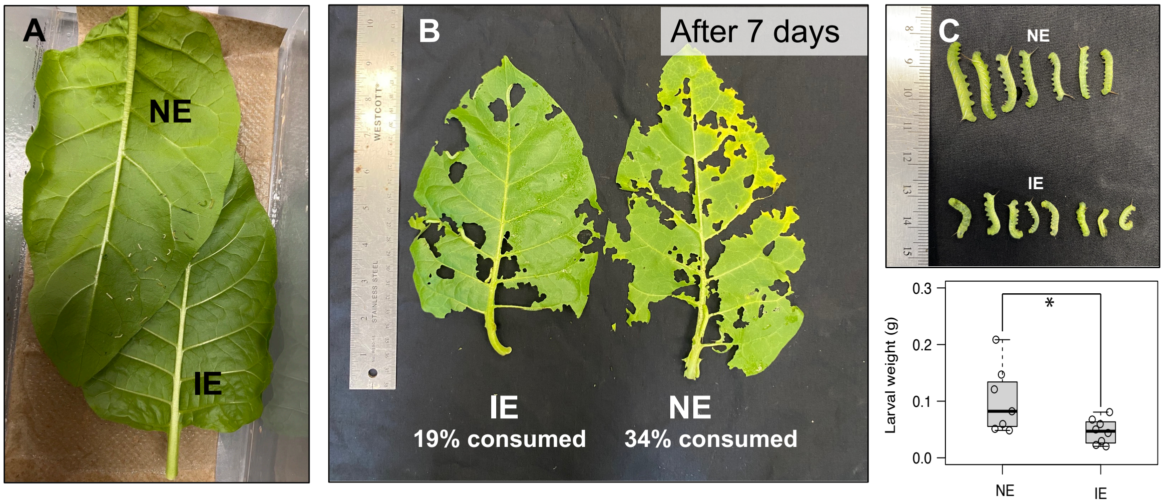

Fig. S1. **Hornworm feeding preference study after hatching. (A)** Worms were reared in a box containing a pair of IE and NE leaves for 7 d. **(B)** Comparison of leaf consumption 7 d post-feeding when given the choice between NE and IE leaves. **(C)** Comparison of hornworm larval weight recovered from IE and NE leaves after 7 d (n=7-8). Asterisk indicates significantly lower larval weight in IE leaves compared to NE leaves 10 d post-feeding (P<0.05; Student's t-test).

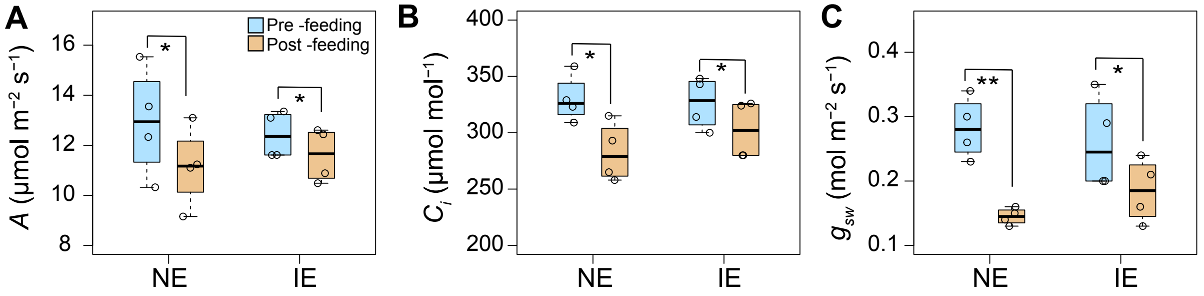

Fig. S2. Comparison of (A) photosynthesis (*A*), (B) intercellular CO_2 concentration (C_i), and (C) stomatal conductance (g_{sw}) in NE and IE leaves pre- and post-feeding. Asterisks indicate significant decrease after 45 min worm feeding (*- *P*<0.01; **- *P*<0.01; Student's t-test). Whiskers of the box plots represent 95% confidence interval.

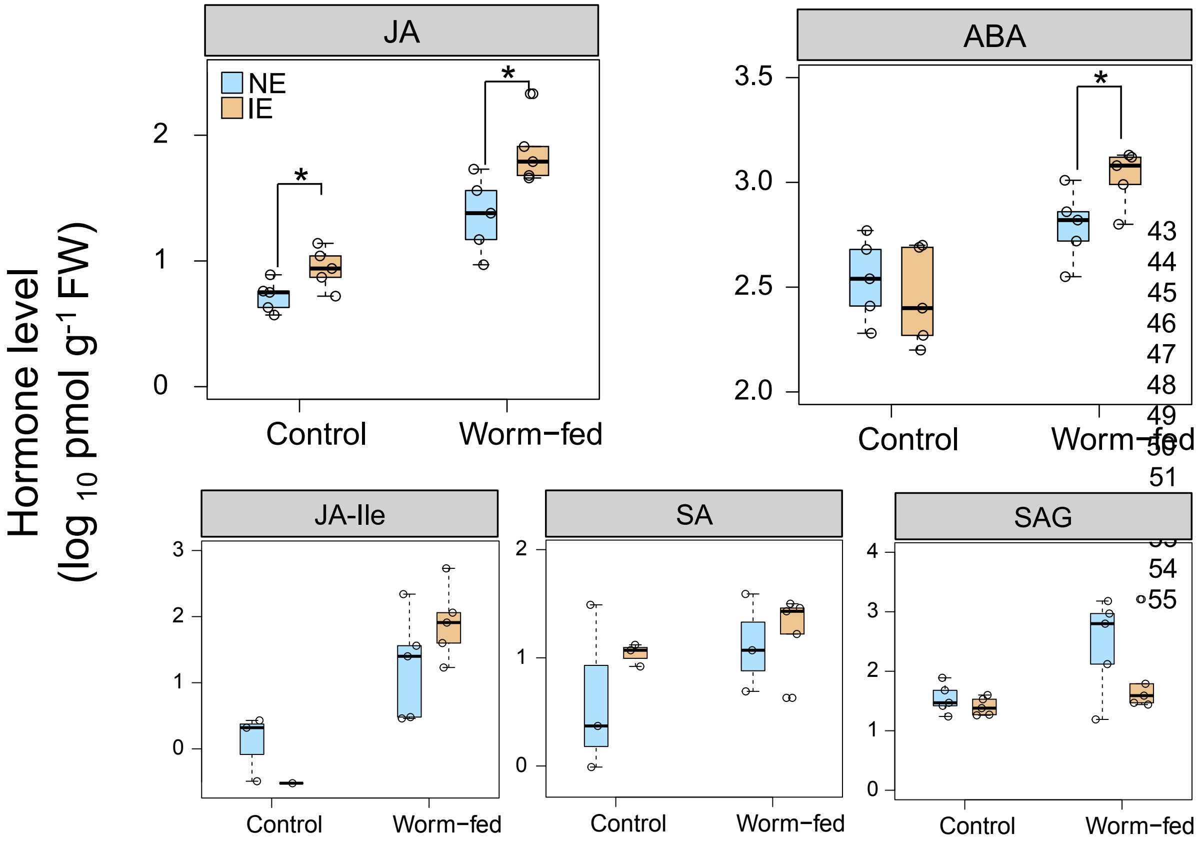

Fig. S3. **Endogenous hormone levels in NE and IE leaves after long-term worm feeding.** Hormones were quantified in leaves 10 d post-feeding (*n*=3-5). Asterisk indicates significantly higher JA and ABA levels in IE leaves post-feeding compared with NE leaves (*P*<0.05; Student's t-test). Change in JA-Ile, SA, and SAG levels between NE and IE lines was not statistically significant. Control plants were never exposed to worms. Whiskers of the box plots represent 95% confidence interval. Abbreviation: SA- Salicylic acid.

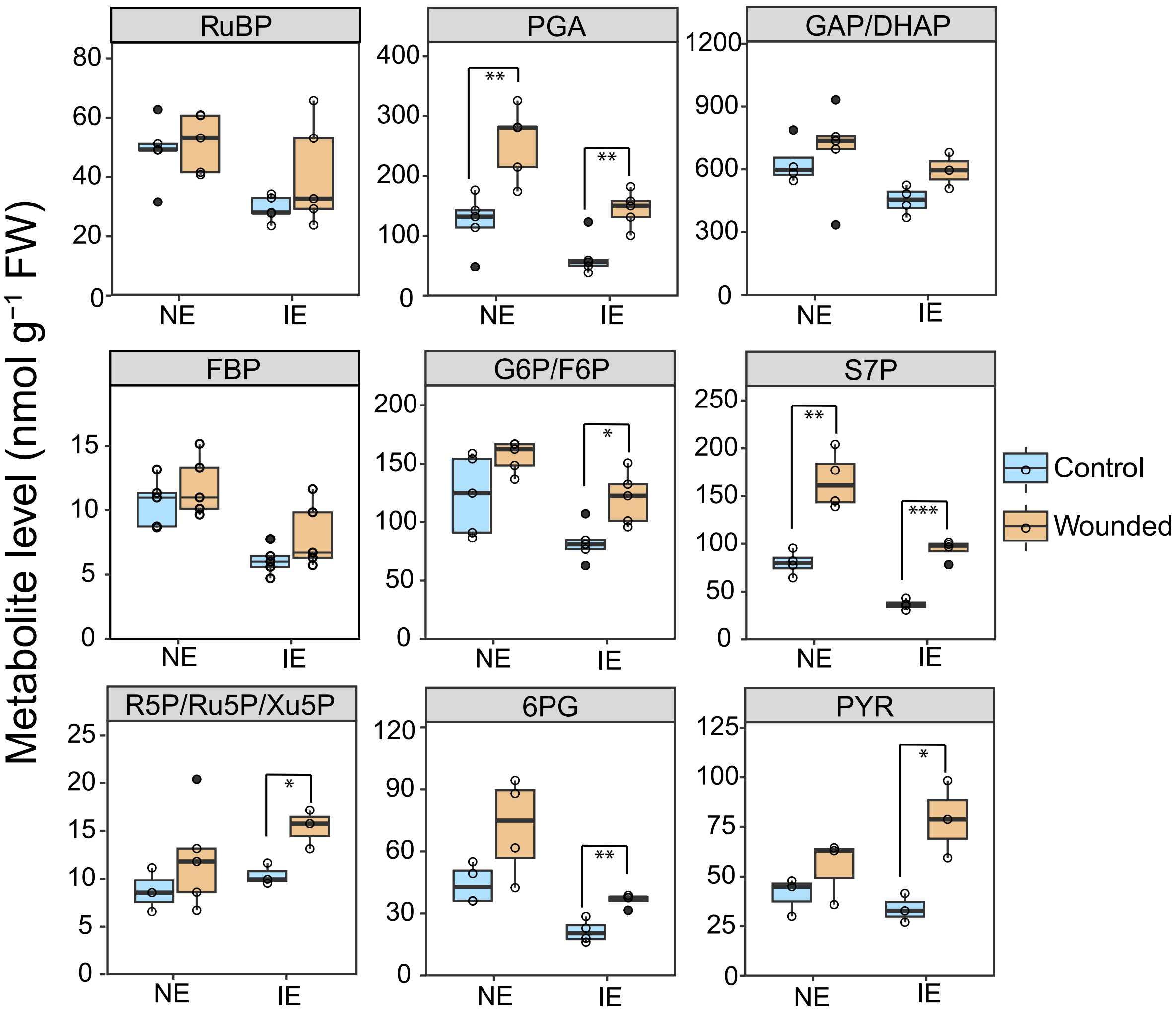

Fig. S4. **Change in CBC metabolites after wounding in NE and IE leaves.** Asterisks indicate significant increase in wounded leaves compared to unwounded (control) leaves (*- *P*<0.01; **- *P*<0.01; ***- *P*<0.001; Student's t-test). Whiskers of the box plots represent 95% confidence interval. Abbreviations: FBP- fructose 6(bis)-phosphate; RuBP- Ribulose 1,5-bisphosphate; PGA- 3-phosphoglycerate; G6P- glucose 6-phosphate; F6P- fructose 6-phosphate; R5P- ribose 5-phosphate; Ru5P, ribulose 5-phosphate; Xu5P- xylulose 5-phosphate; 6PG- 6 phosphogluconate; S7P- sedoheptulose 7(bis)-phosphate; PYR- pyruvate; GAP- glyceraldehyde 3-phosphate; DHAP- dihydroxyacetone phosphate.

	Units	NE	IE
V_{cmax}	$\mu mol\ m^{-2}\ s^{-1}$	73.5 ± 4.9	77.7 ± 9.9
J	$\mu mol\ m^{-2}\ s^{-1}$	116.1 ± 10.9	114.0 ± 4.6
TPU	$\mu mol\ m^{-2}\ s^{-1}$	7.8 ± 0.9	7.4 ± 0.4
R_d*	$\mu mol\ m^{-2}\ s^{-1}$	2.0 ± 0.0	2.0 ± 0.0
g_m	$\mu mol\ m^{-2}\ s^{-1} Pa^{-1}$	1.5 ± 0.3	0.9 ± 0.2
α_G	none	0.0 ± 0.0	0.0 ± 0.0
α_S	none	0.3 ± 0.1	0.4 ± 0.0

Table S1: Photosynthesis and *A/Ci* curve parameters in NE and IE leaves recorded during worm feeding. No significant difference was observed between the NE and IE lines. V_{cmax}– Rubisco capacity; *J*- electron transport; *TPU*- triosephosphate use; R_d- respiration; g_m- mesophyll conductance; α_G- proportion of carbon exported from photorespiration as glycine; α_S- proportion of carbon exported from photorespiration as serine.

Movie S1: Hornworm feeding preference study. Feeding behavior of 3rd instar larvae was monitored when given the choice between NE and IE leaves. Although the worms crawled towards the IE leaf, they turned away and preferred to feed on the NE leaf.
